# Genome Analysis of a Polysaccharide-Degrading Bacterium *Microbulbifer* sp. HZ11 and Degradation of Alginate

**DOI:** 10.3390/md22120569

**Published:** 2024-12-18

**Authors:** Xiao Liu, Wentao Zhao, Yan Li, Zhongliang Sun, Chang Lu, Liqin Sun

**Affiliations:** Yantai Key Laboratory of Characteristic Agricultural Bioresource Conservation & Germplasm Innovative Utilization, School of Life Sciences, Yantai University, Yantai 264000, China; trap987654321@163.com (X.L.); z2952990092@163.com (W.Z.); yanli@ytu.edu.cn (Y.L.); zlsun2015@163.com (Z.S.)

**Keywords:** *Microbulbifer* sp., alginate lyase, polysaccharide lyase, alginate-degrading strain

## Abstract

Marine bacteria are crucial sources of alginate lyases, which play an essential role in alginate oligosaccharide (AOS) production. This study reports the biochemical characteristics of a new species of the *Microbulbifer* genus, *Microbulbifer* sp. HZ11. The strain HZ11 is Gram-negative, aerobic, flagellate-free, and rod-shaped. The genome of strain HZ11 is a 4,248,867 bp circular chromosome with an average GC content of 56.68%. HZ11 can degrade alginate and other polysaccharides. The carbohydrate-active enzyme (CAZyme) genes account for 4.57% of the total protein-coding genes of HZ11. Its alginate metabolism process is consistent with the characteristics of the polysaccharide utilization locus (PUL) system. The alginate lyase produced by strain HZ11 showed the highest activity at 50 °C, pH 8.5, and 0.1 M NaCl. The substrate preference was as follows: sodium alginate > poly mannuronic acid > poly guluronic acid. The thin layer chromatography (TLC) results revealed that the main enzymatic degradation products were monosaccharides or AOSs with a degree of polymerization (DP) of 2–3. These results help clarify the metabolism and utilization mechanism of alginate by marine bacteria and provide a theoretical reference for its application in the degradation of alginate and other polysaccharides.

## 1. Introduction

Alginate is a linear copolymer composed of α-L-guluronic acid (G) and β-D-mannuronic acid (M) linked by (1→4) glycosidic bonds in different proportions [1]. As a structural polysaccharide in the cell walls of brown algae, alginate is highly important for maintaining the structural morphology of brown algae cells and resisting external mechanical shearing [2]. In addition, alginate is also important in the industrial, food, and medical fields [3].

The molecular weights of commercially available alginates are mostly 33,000–400,000 Da, indicating slow dissolution rates, high solution viscosity, and difficulty penetrating biological membranes for absorption and utilization by the organism [4,5]. However, alginate oligosaccharides (AOSs) with a degree of polymerization (DP) of 2–25 after degradation tend to exhibit better physicochemical properties and greater biological activities, such as anti-inflammatory, antioxidation and antitumor effects [6]. The DP and the composition ratio of M/G affect the biological activities of AOSs [7,8,9]. Therefore, efficiently and directionally preparing AOSs is critical in its research and utilization.

Compared with traditional physical and chemical degradation methods, enzymatic degradation under mild conditions results in high yields and products that are easy to separate and purify, representing an efficient and environmentally friendly method for preparing AOSs [10]. Alginate lyases act on alginate glycosidic bonds through a β-elimination reaction, causing chain dehydration to form double bonds and generating unsaturated oligosaccharide molecules [11]. These genes are divided into 14 polysaccharide lyase (PL) families on the basis of amino acid sequence similarity (the PL5, PL6, PL7, PL8, PL14, PL15, PL17, PL18, PL31, PL32, PL34, PL36, PL39, and PL41 families). The protein structures include β-jelly roll folds (PL7, PL14, PL18 and PL36 as representatives), β-helix folds (PL6 and PL31), (α/α)_n_ Toroid folds (PL5), and (α/α)_n_ toroid + β-jelly roll folds (PL15, PL17 and PL39) [12]. The action modes are divided into endo-type and exo-type, and most of the alginate lyases known are endoenzymes that act on the internal sugar chain to produce oligosaccharides with different DPs; only a few are exoenzymes that cleave the substrate molecule from one end, producing monosaccharides. These enzymes can be divided into alginate lyases that specifically degrade poly galacturonic acid (polyG) (EC 4.2.2.11), alginate lyases that specifically degrade poly mannuronic acid (polyM) (EC 4.2.2.3), and bifunctional alginate lyases (EC 4.2.2-) [13].

However, the high price and low output of alginate lyase preparations have restricted the promotion of this method and its large-scale application. Therefore, exploring more highly efficient sources of alginate lyase is important for achieving an efficient and green industrial production of AOSs. Recent studies have focused on the isolation, characterization, and production of alginate lyases. In addition to marine fungi, marine bacteria isolated from rotten seaweed, marine mollusks, marine sediments, or seawater also represent a significant source of these enzymes [14,15,16]. More than 50 species of marine bacteria capable of producing alginate lyases have been identified, including *Pseudomonas* sp., *Flavobacterium* sp., *Pseudoalteromonas* sp., *Microbulbifer* sp. and *Vibrio* sp., among others [17,18,19,20,21]. These marine bacteria employ polysaccharide utilization loci (PUL) systems, “scattered” systems, or “pit” transport mechanisms to degrade alginate into oligosaccharides, which are subsequently transported into the cells for further degradation and metabolism as their growth carbon source [22].

At present, many alginate lyase genes have been successfully heterogeneously expressed, purified, and characterized, especially the members from the PL6, 7 and 17 families [23,24,25]. In order to optimize the catalytic performance of recombinant enzymes, improve their stability, or broaden their substrate range, rational and semi-rational structural modifications of alginate lyases have been carried out based on their three-dimensional structure and amino acid sequence, such as site-directed mutagenesis and domain truncation, effectively improving the efficient catalytic performance and application potential of recombinant alginate lyase in alginate degradation [26]. Compared with recombinant alginate enzymes, directly utilizing crude extracellular enzymes in research can significantly reduce the costs associated with enzyme preparations while minimizing activity losses during purification processes [27]. Crude enzymes have been extensively employed to investigate enzymatic efficiency, kinetics and thermal stability, among other parameters [28,29].

*Microbulbifer* sp. is a kind of halophilic marine bacteria known for their excellent polysaccharide degradation ability [30]. At present, research on the production of alginate lyase by the genus *Microbulbifer* mainly focuses on the heterologous expression of alginate lyase genes and characterization of recombinant enzymes, such as AlgL6 (from *M.* sp. ALW1), MiAly17A (from *M. arenaceous*), and AlgSH17 (from *M.* sp. SH-1) [23,24,25]. Alternatively, *M.* sp can be used to hydrolyze seaweed [31,32]. However, there has been limited in-depth research on the alginate metabolism pathway and production of alginate lyase in *M.* sp. In this study, we investigated a marine bacterium *Microbulbifer* sp. HZ11 that produces extracellular alginate lyase. Research elucidated the metabolic pathway for alginate in HZ11, and characterized the enzymatic properties of its extracellular alginate lyase. The results will provide valuable resources for future preparation and application studies involving alginate lyases and AOSs.

## 2. Results

### 2.1. Characterization and Systematic Classification of Strain HZ11

**Figure 1:**
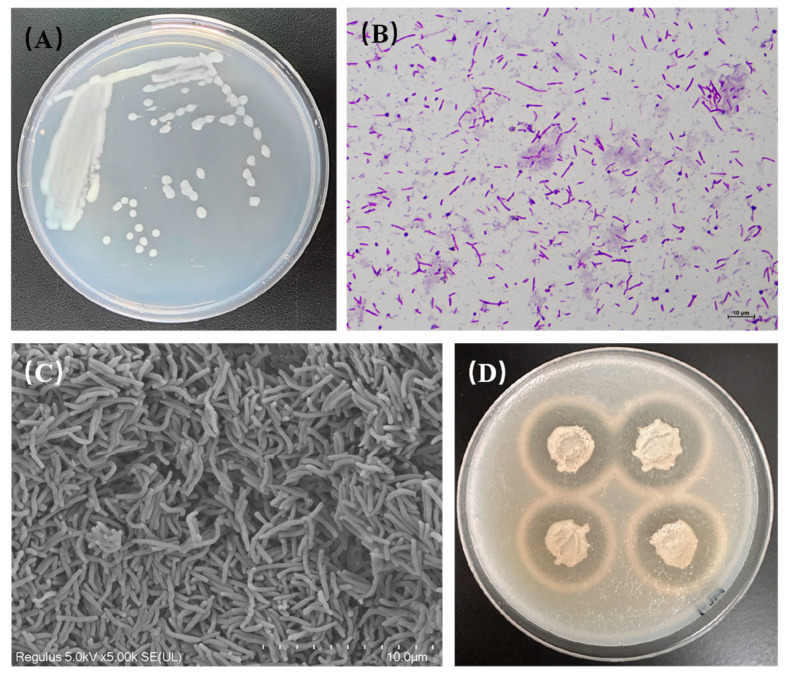
Morphological observation and identification of the alginate lyase production ability of strain HZ11. (**A**) Colony morphology. (**B**) Gram staining (1000×). (**C**) Scanning electron microscope image (10,000×). (**D**) Growth state of strain HZ11 on a marine basal culture medium plate containing 0.5% alginate, with a transparent hydrolysis zone around the colony after treatment with 1 M calcium chloride solution.

After incubation at 30 °C on a 2216E plate for 48 h, the HZ11 colony appeared white, smooth, and opaque with a neat edge and a diameter of approximately 2 mm (Figure 1A). The cells of strain HZ11 were Gram-stain-negative (Figure 1B), aerobic, flagellate-free, and nonmotile, with a slender rod-like shape, approximately 0.3–0.5 μm wide and 3–5 μm long (Figure 1C). After incubation on a marine basal culture medium plate containing 0.5% alginate at 30 °C for 48 h, the area surrounding the HZ11 colony exhibited a transparent zone after being treated with 1 M calcium chloride solution (Figure 1D), indicating that HZ11 could produce exocellular alginate lyase.

The results of the physiological and biochemical identification of strain HZ11 are shown in Table 1. HZ11 can grow in the temperature range of 15–50 °C, pH range of 3.0–9.5, and sodium chloride concentration range of 20–90 g/L, with an optimal growth temperature of 28–30 °C, optimal pH of 8.5, and optimal sodium chloride concentration of 70 g/L.

**Figure 2:**
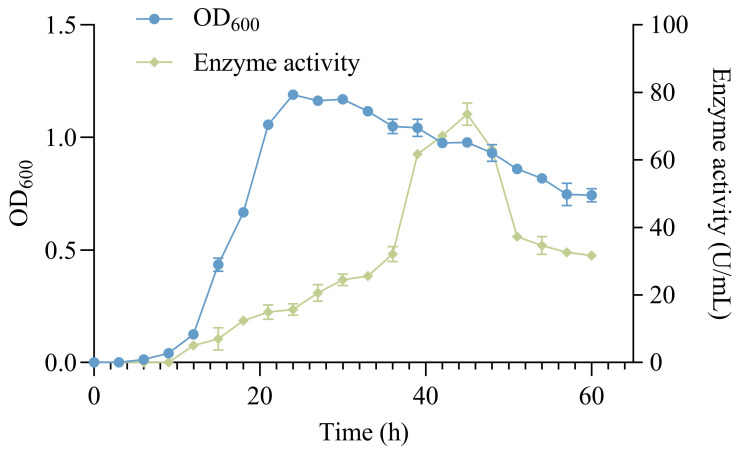
HZ11 growth and enzyme production curve in marine basal culture medium.

The 2% HZ11 bacterial suspension was added to the marine basal culture medium. The culture was carried out at 25 °C with a shaking speed of 120 rpm. After 6 h, HZ11 entered the logarithmic growth phase, and the OD_600_ increased rapidly, reaching its highest value after 24 h. After 12 h of inoculation, the alginate lyase enzyme activity in the fermentation mixture was detected, and the enzyme activity reached its highest level of 73.58 ± 3.29 U·mL^−1^ after 45 h of culture (Figure 2).

**Figure 3:**
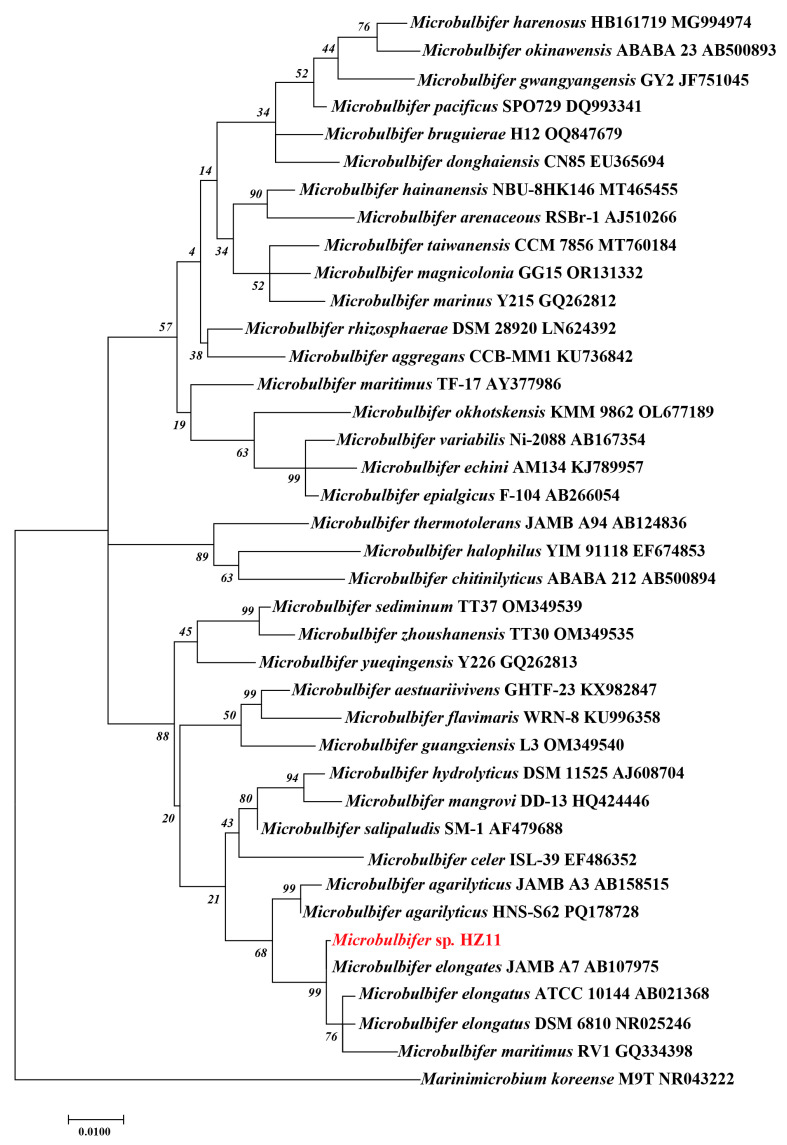
Maximum likelihood phylogenetic tree based on the 16S rRNA gene of *Microbulbifer* genus bacteria. *Marinimicrobium koreense* M9T was used as an outgroup.

An amplification of the 16S rRNA gene from the HZ11 genome resulted in a sequence of 1412 bp (NCBI accession number: PQ339657). The NCBI database comparison revealed that the sequence had the highest similarity with the 16S rRNA gene of *Microbulbifer elongatus* JAMB A7 (99.93%), followed by *M. maritimus* RV1 (98.66%), *M. agarilyticus* HNS-S62 (98.23%), and *M. guangxiensis* L3 (98.02%). A phylogenetic tree was constructed on the basis of the 16S rRNA gene sequence, and strain HZ11 clustered within the *M. elongatus* branch (Figure 3).

**Figure 4:**
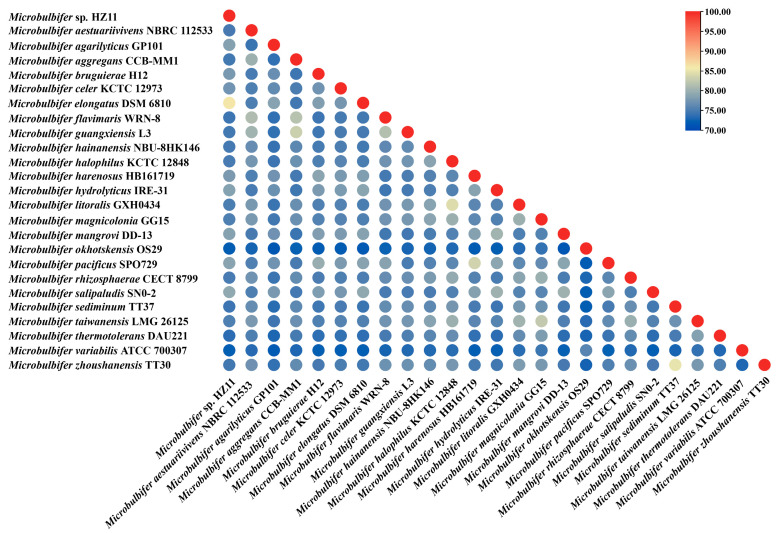
ANI heatmap of the genomes of HZ11 and other *Microbulbifer* strains.

To further clarify the taxonomic status of HZ11, ANI (Figure 4) and dDDH (Table S1) were calculated on the basis of the genomic data of HZ11 and other *Microbulbifer* species. HZ11 presented the highest ANI value of 86.19% with *M. elongatus* DSM 6810, followed by *M. salipaludis* SN0-2 and *M. pacificus* SPO729, with ANI values of 79.3% and 78.85%, respectively. The ANI values for *M. agarilyticus* GP101 and *M. guangxiensis* L3 were 78.52% and 74.74%, respectively (*M. maritimus* RV1 does not have published genomic data and could not be compared). Similarly, HZ11 had the highest dDDH value with *M. elongatus* DSM 6810 at 30%, followed by *M. salipaludis* SN0-2 at 23.4% and *M. mangrovi* DD-13 at 23%.

### 2.2. Genome Annotation and Analysis

**Figure 5:**
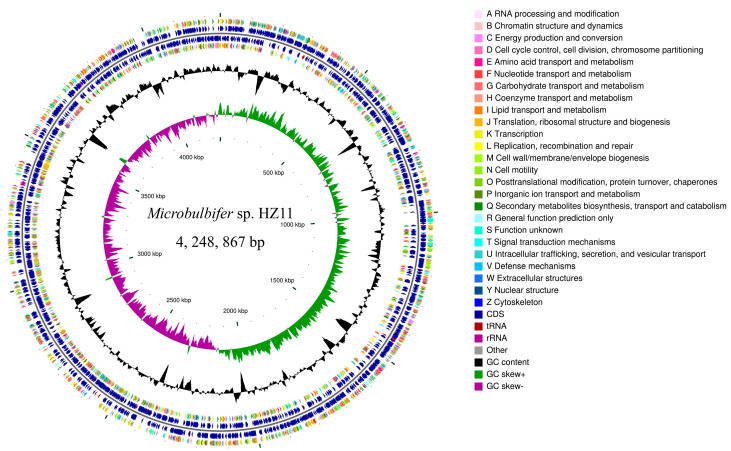
Circle diagram of the HZ11 genome, from the outside to the inside: the first and fourth circles are CDSs on the positive and negative strands, with different colors representing different COG functional classifications; the second and third circles are CDSs, tRNAs, and rRNAs on the positive and negative strands; the fifth circle represents the GC content; the sixth circle represents the GC-skew value; and the innermost circle represents the size identification of the genome.

Complete genome sequencing of HZ11 was performed, and one circular chromosome was identified (Figure 5) (NCBI: PRJNA1153437). A total of 219,090 reads were analyzed, with an average read length of 7809.67 bp. The genome size of HZ11 was approximately 4,248,867 bp, with a GC content of 56.68%. A total of 3477 protein-coding sequences were predicted, with a total gene length of 3,733,023 bp and an average of 1073.63 bp. The average gene density was 0.82, with an average GC content of 57.40%. There were 84 repetitive sequences, 48 tRNA sequences, 12 rRNA sequences, and 14 sRNA sequences (Table 2).

By classifying genes on the basis of their functions via the Cluster of Orthologous Groups of Proteins (COG) and KEGG databases, a total of 2863 genes were found to have COG annotations, which were grouped into 24 classes, accounting for 82.34% of the total genes. These genes were enriched mainly in [E] (amino acid transport and metabolism, 237), [J] (translation, ribosome structure and biogenesis, 246), [M] (cell wall/membrane/envelope biogenesis, 255), and [P] (inorganic ion transport and metabolism, 229).

A total of 2051 genes were annotated to the KEGG database, accounting for 58.98% of all genes. These genes were divided into six major classes according to metabolic pathways and 42 smaller classes according to specific functions. Among them, metabolism-related genes were the most abundant, totaling 1641, accounting for 47.19% of all coding genes in the genome. These genes control nucleic acid, amino acid, carbohydrate, and energy metabolism. In addition, 208 genes encoding genes involved in environmental information processing, including those involved in signal transduction and transport functions of the cell membrane, were identified.

### 2.3. The Utilizing Abilities of Carbohydrates

**Figure 6:**
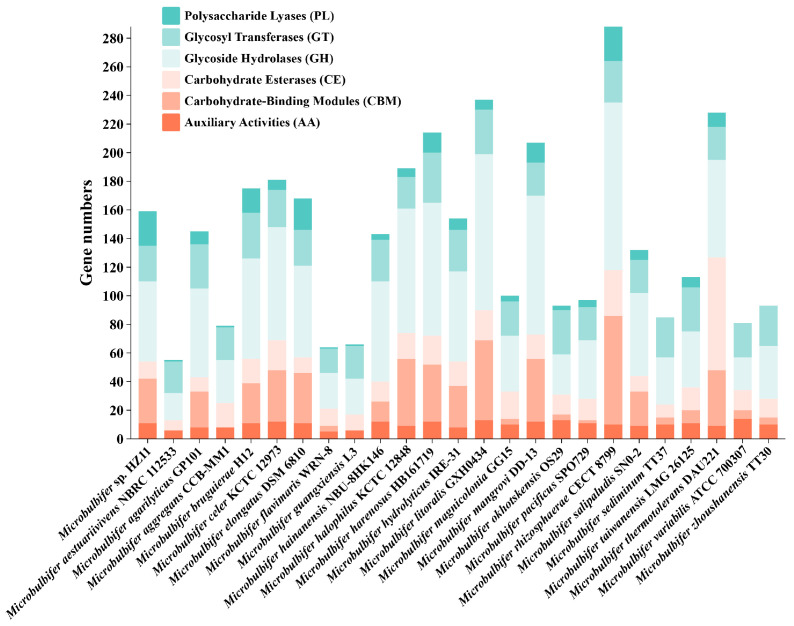
Number of carbohydrate-active enzyme-encoding genes classified by the CAZyme database for each strain.

To better explore carbohydrate utilization and degradation-related enzymes in the HZ11 genome, the CAZyme database was used to annotate the coding sequences of HZ11. A total of 159 CAZymes were identified in HZ11, accounting for 4.57% of the total protein-coding genes, including auxiliary activities (AA, 11), carbohydrate-binding module (CBM, 31), carbohydrate esterases (CE, 12), glycoside hydrolases (GH, 56), glycosyl transferases (GT, 25), and polysaccharide lyases (PL, 24). PL and GH, the leading CAZyme families involved in polysaccharide degradation, accounted for 50.31% of the CAZyme family genes (Figure 6).

As shown in Table 3, the PL genes belong to seven families (PL1, PL10, PL6, PL7, PL17, PL18, and PL38), which are related mainly to pectin and alginate degradation. A total of 10 alginate lyase sequences were annotated (PL7: gene0073, 0074, 0628, 1910, 1911, 3508; PL6: gene0081; PL17: gene0080; PL18: gene0070; PL38: gene0297). The remaining eight sequences contained signal peptides, with the exception of genes0073 and 3508. Gene0070, 0074, 0628, 1910 and 1911 contained CBM32 or CBM16 domains.

Four cellulase genes belonging to the GH5 (gene1049, gene1101, gene2339) and GH9 (gene1102) genes are modular enzymes, with the CBM2, CBM3, CBM6, and CBM16 structural domains observed. Five α-amylase genes (gene1263, gene3041, gene3042, gene3044, and gene3048), one pullulanase gene (gene0349), and one neopullulanase gene (gene3042) belonging to the GH13 family were identified. Four chitinase genes (gene1079, gene1080, gene2143, and gene2144) belonging to the GH18 family were also identified, mainly with the CBM73 domain. In addition, genes encoding enzymes belonging to the GH10 (Endo-1,4-β-xylanase), GH13 (α-glucosidase), GH3 (β-glucosidase), GH2 and GH167 (β-galactosidase) families were also identified.

**Figure 7:**
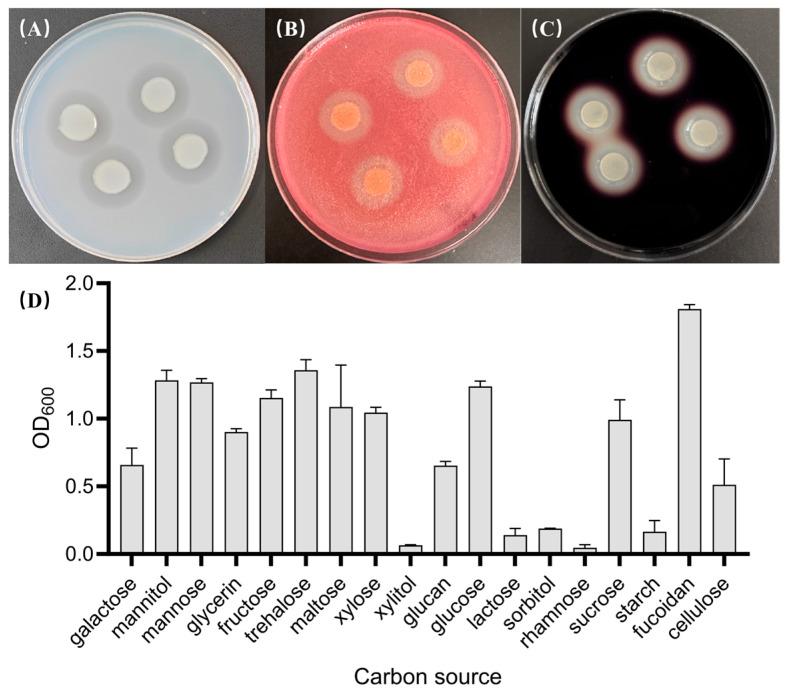
HZ11 carbohydrate utilization detection. (**A**) Growth of HZ11 on a marine basal culture plate with agar as the carbon source, with a transparent hydrolysis zone around the colony. (**B**) The growth of HZ11 on marine substrate plates containing cellulose, after being stained with 1 mg/mL Congo red solution, resulted in a transparent hydrolysis zone around the colony. (**C**) Growth of HZ11 on a marine-based culture plate containing soluble starch after being stained with Lugol’s iodine, with a transparent zone of hydrolysis around the colony. (**D**) Growth status of HZ11 using different carbohydrates or alcohols as carbon sources.

Under the action of such a complex carbohydrate metabolism system, in addition to alginate, HZ11 also exhibited significant degradation ability for a variety of terrestrial and marine polysaccharides, such as agar, cellulose, and starch (Figure 7A–C). In addition, HZ11 can grow via the use of a variety of monosaccharides, disaccharides, or alcoholic substances as carbon sources (Figure 7D).

**Figure 8:**
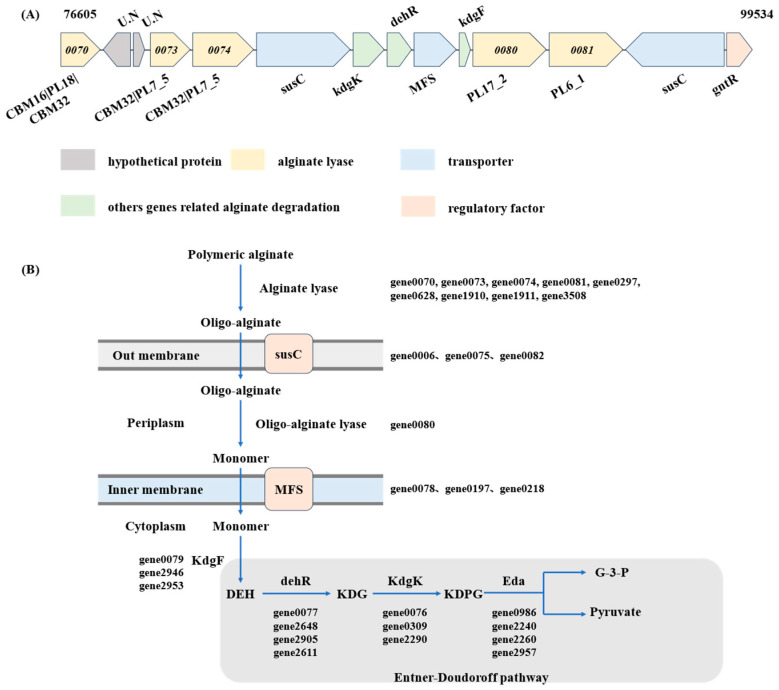
Degradation and utilization pathway of alginate by HZ11. (**A**) Alginate utilization loci of HZ11. (**B**) Alginate metabolic pathway of HZ11. susC, TonB-dependent receptor; kdgK, 2-dehydro-3-deoxygluconokinase; dehR, 4-deoxy-L-erythrohexoseulose uronicacid reductase; MFS, hexuronate transporter; kdgF, pectin degradation protein; gntR, transcriptional regulator; U.N, unknown protein. DEH, 4-deoxy-L-erythro-hexoseulose uronic acid; KDG, 2-keto-3-deoxy-D-gluconate; KDPG, 2-keto-3-deoxy-6-phospho-gluconate; Eda, KDPG aldolase; G-3-P, glyceraldehyde-3-phosphate.

In the genomes of alginate-degrading bacteria, there is often one or more alginate utilization loci (AULs), as well as regulatory elements and transport systems involved in oligosaccharide binding and uptake. The annotation and PUL search results revealed a polysaccharide utilization locus related to alginate in the HZ11 genome (Figure 8A). The alginate degradation enzyme-encoding genes gene0070, gene0073, gene0074, gene0080 and gene0081 are located in a large gene cluster of 14 genes (22.9 kbp). The gene cluster contained vital enzyme-encoding genes involved in alginate metabolism: 2 TonB-dependent receptor (susC) genes (gene0075 and gene0082), a 2-dehydro-3-deoxygluconokinase (gene0076) gene, a dehR gene (gene0077), a hexuronate transporter (MFS) gene (gene0078), and a pectin degradation protein (kdgF) gene (gene0079).

**Figure 9:**
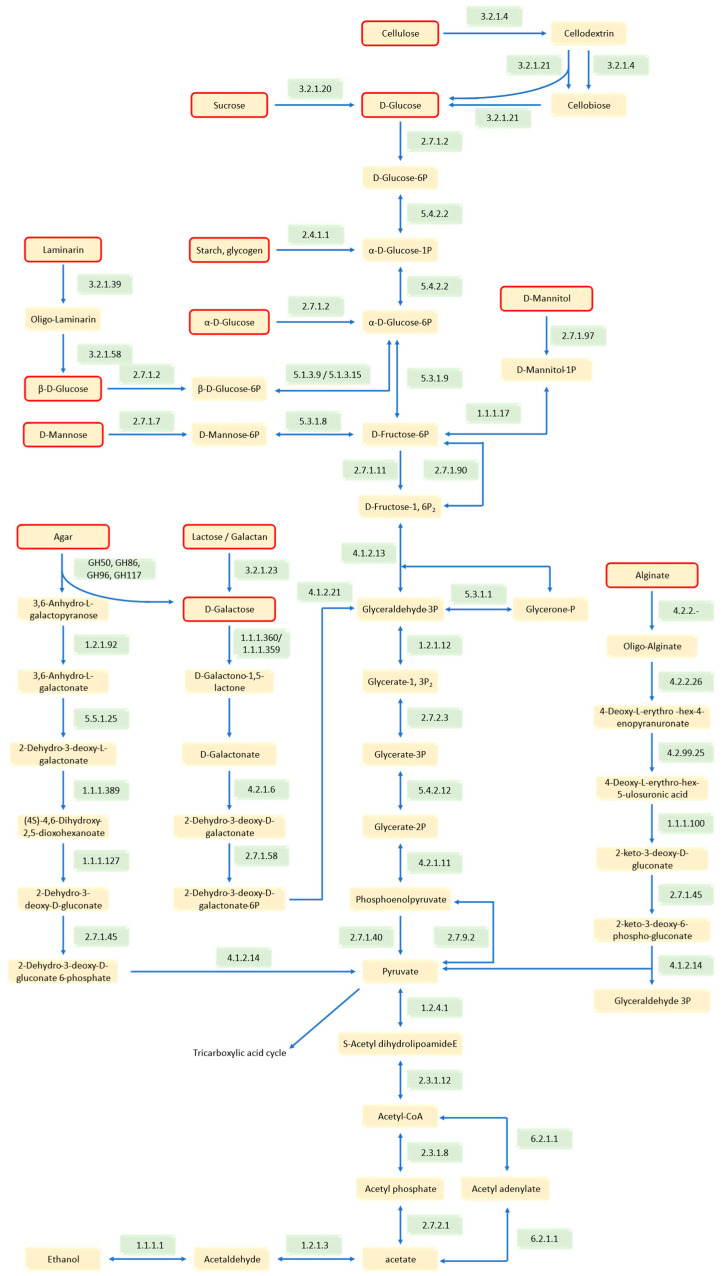
Different carbohydrate metabolic pathways in HZ11 cells. The compounds marked in the red box are the metabolizable carbon sources of HZ11. The numbers in the green box are the EC numbers of the enzymes.

The alginate metabolic pathway of HZ11 fits the characteristics of the AUL system (Figure 8B and Figure 9). In this metabolic process, high-molecular-weight alginate is first broken down into AOSs by alginate lyase secreted by HZ11, which is transported by susC into the periplasm. In the extracellular matrix, AOS is hydrolyzed into unsaturated monosaccharides by oligo alginate lyase. A gene encoding an alginate oligosaccharide lyase (gene0080) was identified in the HZ11 genome. Bioinformatics analysis revealed that the gene contained a signal peptide and transmembrane helix domain, suggesting that it was located in the peripheral cytoplasm, which is consistent with the characteristics of the PUL system. The MFS transported the monosaccharides from the inner membrane space to the cytoplasm, where the unsaturated monosaccharides were converted into open-ring alduronic acid by kdgF. The 4-deoxy-L-erythro-hexoseulose uronic acid (DEH) was formed and reduced to 2-keto-3-deoxy-D-gluconate (KDG) by DEH reductase. KDG kinase (kdgK) then converts KDG into 2-keto-3-deoxy-6-phospho-gluconate (KDPG), which is split into glyceraldehyde-3-phosphate (G3P) and pyruvate by KDPG aldolase (Eda) and enters the tricarboxylic acid (TCA) cycle.

Using the KEGG database, a metabolic pathway for the utilization of other carbohydrates, such as starch, glucose, and cellulose, by HZ11 was constructed (Figure 9). The results revealed that HZ11 has a complete Entner–Doudoroff (ED) pathway in its genome. Different carbohydrates generate pyruvate through the ED pathway, which enters the TCA cycle. In addition, there was a complete metabolic pathway within the cytoplasm of HZ11 for converting pyruvate to ethanol. Two key enzymes, pyruvate decarboxylase (EC 1.2.4.1) and alcohol dehydrogenase (EC 1.1.1.1), were found.

### 2.4. Detection of Alginate Lyase Activity and Enzymatic Properties

**Figure 10:**
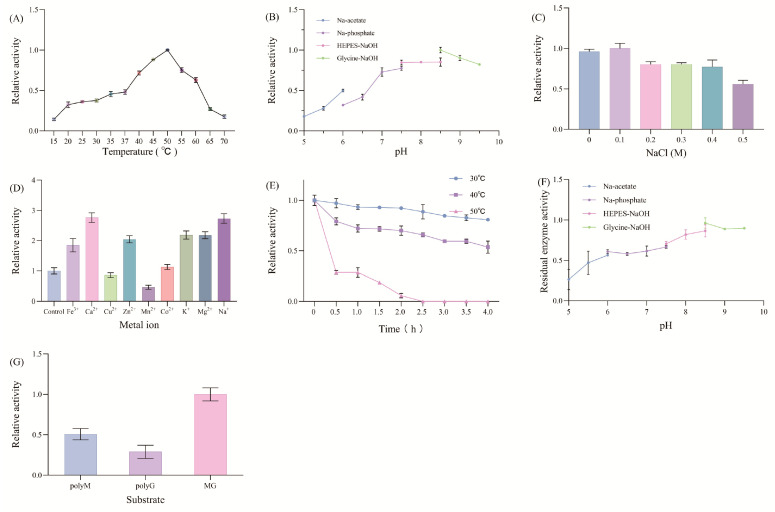
HZ11 alginate lyase enzymatic parameters. (**A**) Optimal temperature. (**B**) Optimal pH. (**C**) Concentration of sodium chloride. (**D**) Effects of metal ions on enzyme activity. (**E**) Thermal stability. (**F**) pH stability. (**G**) Substrate specificity.

The effects of temperature, pH, and sodium chloride concentration on the activity of alginate lyase from brown algae were investigated. As shown in Figure 10A, the optimal enzymatic hydrolysis temperature was 50 °C. In the 35–60 °C temperature range, the enzyme activity was at least 50% of its maximum. The enzyme had deficient activity at temperatures below 15 °C or above 70 °C. The optimal pH was 8.5. Enzyme activity rapidly diminished when the pH fell below 7 (Figure 10B). The optimal sodium chloride concentration was 0.1 M (Figure 10C). Compared with those at high-salt concentrations, the enzymes at low-salt concentrations presented greater enzymatic activity. When 5 mM of metal ions were added to the system, Fe^3+^, Ca^2+^, Zn^2+^, Co^2+^, K^+^, Mg^2+^, and Na^+^ enhanced the enzymatic activity, with Ca^2+^ showing the most significant increase, being 2.76-fold greater than that of the control group, followed by Na^+^, which was 2.73-fold greater than that of the control group. The enhancement effect of Co^2+^ was relatively weak, being 1.13-fold greater than that of the control group. Cu^2+^ inhibited the enzyme slightly, with the enzymatic activity being 98% that of the control group. Mn^2+^ had a more significant inhibitory effect, with a value of only 58% in the control group (Figure 10D).

The enzyme had a certain degree of thermal stability, with 80.8% and 53.6% of the original enzymatic activity remaining after incubation at 30 °C and 40 °C for 4 h, respectively. After incubation at 50 °C for 0.5 h, the enzymatic activity decreased significantly, with only 26.6% of the original activity remaining. After incubation for 2.5 h, the enzymatic activity was completely lost (Figure 10E). The enzymes were relatively stable at pH 5.5–9.5, which retained more than 60% of the enzyme activity after incubation at alkaline pH for 24 h (Figure 10F). The substrate preference of HZ11 alginate lyase was alginate > polyM > polyG, with the enzymatic activities of polyM and polyG relative to alginate being 50.7% and 28.8%, respectively (Figure 10G).

**Figure 11:**
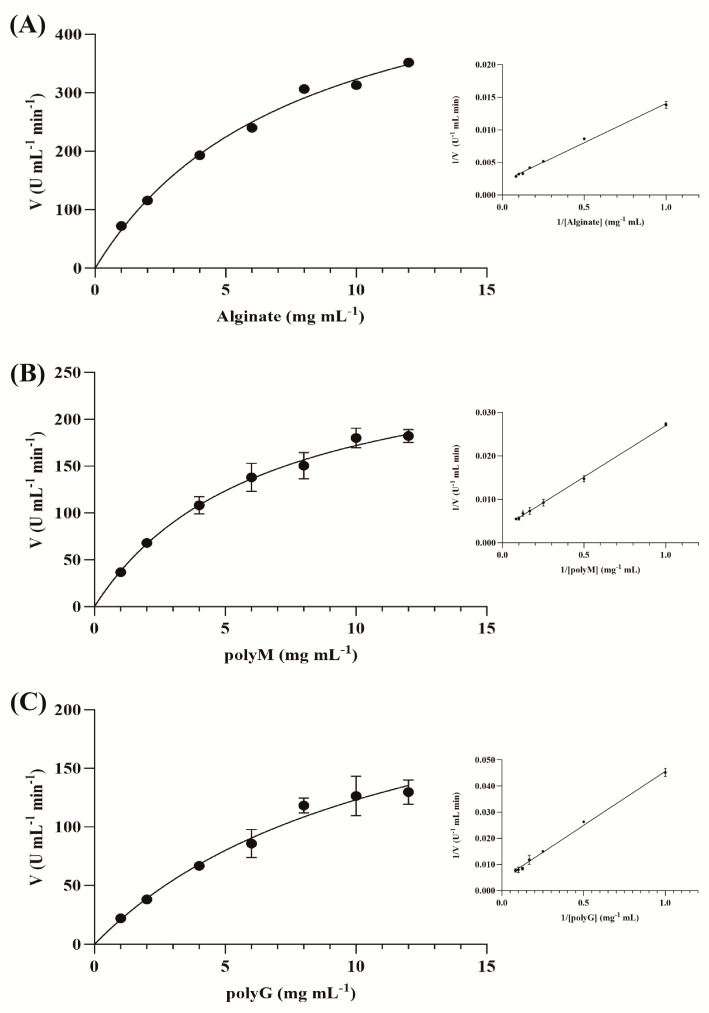
The Michaelis–Menten graph and Lineweaver–Burk plots of the HZ11 extracellular alginate lyase for different substrates; (**A**) alginate as a substrate; (**B**) polyM as a substrate; (**C**) polyG as a substrate.

The Km of polyM, polyG and alginate were 6.95 ± 0.15 mg mL^−1^, 9.53 ± 0.23 mg mL^−1^ and 5.95 ± 0.12 mg mL^−1^, respectively, and the Vmax were 294.8 ± 17.08 U mL^−1^ min^−1^, 231.0 ± 21.11 U mL^−1^ min^−1^ and 494.8 ± 26.22 U mL^−1^ min^−1^ (Figure 11, Table 4).

**Figure 12:**
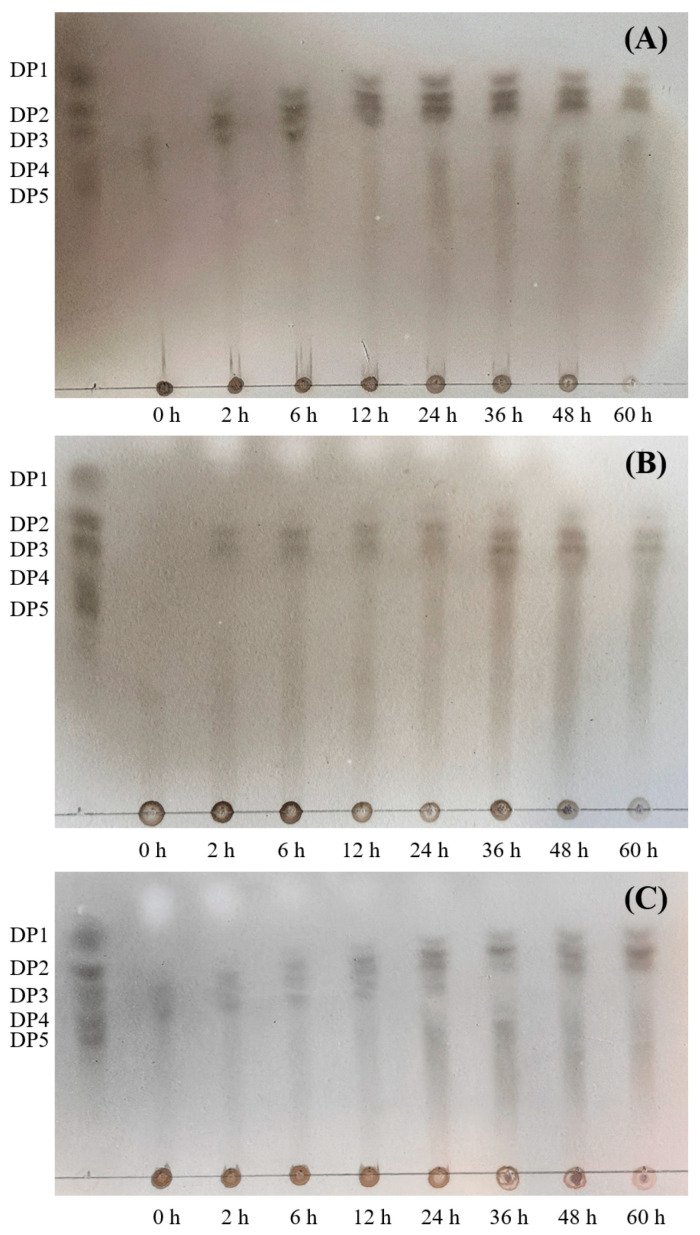
TLC analysis of the HZ11 alginate lyase degradation products; (**A**) alginate as a substrate; (**B**) polyM as a substrate; (**C**) polyG as a substrate.

The products of the enzymatic hydrolysis of HZ11 alginate lyase were analyzed via TLC (Figure 12). AOSs could be detected after 2 h of enzymatic hydrolysis. The degradation products of alginate and polyG were monosaccharides and disaccharides, whereas the degradation products of polyM were mainly AOSs with DPs of 2–3.

### 2.5. RT-qPCR of Alginate Lyases

**Figure 13:**
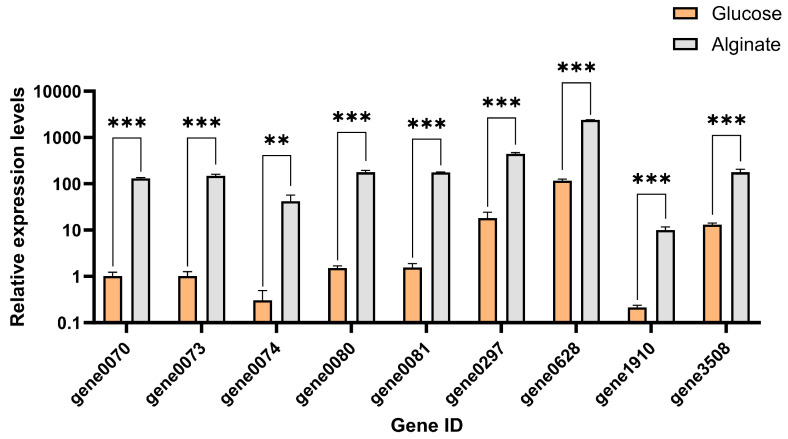
Expression levels of alginate lyase genes in strain HZ11 after 24 h culture. ** *p* < 0.01, and *** *p* < 0.001.

In the cultivation process with alginate as the carbon source, compared with glucose, 9 out of the 10 predicted alginate lyase genes were significantly upregulated (gene 1911 had low expression and could not be effectively detected). The expression of gene 0073 was upregulated the most, at 144.6-fold greater than that in the control group, followed by the expression of genes 0074 (138.7-fold) and gene 0070 (128-fold) (Figure 13).

## 3. Discussion

Marine microorganisms are one of the essential sources of alginate lyase. Researchers have been actively exploring bacterial strains with high efficiency and stability in producing enzymes, thus promoting the scaled production of AOSs by enzymatic hydrolysis. In this study, a marine bacterium HZ11 was researched, which could produce alginate lyase and other extracellular polysaccharides degrading enzymes, including its physiological and biochemical characteristics, genomic data, carbohydrate metabolic pathways and extracellular alginate lyase characteristics.

### 3.1. HZ11 Could Be a New Species of Microbulbifer sp.

As a halophilic bacterium, HZ11 is rod-shaped, aerobic and Gram-negative, exhibiting typical physiological and ecological characteristics of the genus *Microbulbifer*. *Microbulbifer* sp. could produce cellulase, amylase, agarase, chitinase, alginate lyase and xylanase to degrade and utilize these polysaccharides [30]. These bacteria are recognized for their ability to degrade polysaccharides and other polymeric materials. In this study, the ability of HZ11 to degrade different polysaccharides and its utilization capacity had been confirmed through plate experiments and bacterial growth experiments.

The results of 16S rRNA alignment and phylogenetic tree analysis indicated that the strain HZ11 belongs to the genus *Microbulbifer*. Compared with genomic data of other *Microbulbifer* strains, the ANI and dDDH data analysis results showed that the values of HZ11 were under the species boundary thresholds of 95–96% and 70%, respectively [33,34]. In addition, HZ11 also exhibited specific physiological and ecological differences from *Microbulbifer* sp. For example, in the phylogenetic tree, HZ11 and *M. elongatus* have shown recent evolutionary relationships. The optimal growth concentration of NaCl for model strain *M*. *elongatus* DSM 6810^T^ is 2–3%, and negative for urease and nitrate reduction, while HZ11 is 7% and positive. In addition, the optimal growth pH for various known *M.* sp. is 7, while HZ11 is 8.5 [35,36]. Therefore, HZ11 may be a new species of the *Microbulbifer* genus [37].

### 3.2. The Enzyme Properties of HZ11 Was Different with Other M. sp.

Comparing the growth curve and enzyme production curve of HZ11, there was a certain lag in enzyme production, resulting in the highest enzyme activity during the decline phase. This may be due to limited nutrient content (carbon and nitrogen source concentrations were only 0.5%), which reduced the production of extracellular enzymes and prioritized the growth of bacterial cells. During the decline phase, the bacterial cells lysed, releasing intracellular substances and increasing enzyme activity [38].

In the follow-up study, improving the enzyme production efficiency of HZ11 by adjusting the culture conditions (temperature, pH, etc.) or optimizing the medium composition can be further studied.

HZ11 extracellular alginate lyase showed the highest enzyme activity at 50 °C, which was the same as *M. thermocotolerans* HB226069. Although HZ11 is most suitable for high reaction temperatures, its tolerance to high temperatures is poor. After incubation at 50 °C for 30 min, the residual enzyme activity was 30%. However, when *M. thermocotolerans* HB226069 was incubated at 50 °C or even higher for 1 h, at least 40% of the enzyme activity remains. In addition, HZ11 exhibited a pronounced alkaline preference, with the highest enzyme activity and stability at pH 8.5. However, for *M. thermometers* HB226069, they were significantly reduced in alkaline environments [29].

Multiple salt-activated alginate lyases have been reported, such as algl17 (from *M.* sp. ALW1), whose enzyme activity increased by 1.7 times at a NaCl concentration of 0.7 M, demonstrating a certain salt dependence [39]. For HZ11, excessive concentration of NaCl instead inhibits enzyme activity. It is indicated that HZ11 alginate lyase is sensitive to high-salt concentrations and is suitable for salt-free or low-salt environments.

### 3.3. HZ11 Metabolizes Alginate Through the PUL System and Has the Potential to Ferment Brown Algae to Produce Ethanol

Even though many *Microbulbifer* strains have been found to have the ability to degrade alginate, more in-depth investigations and research into their enzyme-producing characteristics and metabolic mechanisms are needed. Alginate lyase bacteria utilize alginate mainly through three different pathways: the polysaccharide utilization loci (PUL) system, the “scattered” system, and the “pit” transport system. The difference between the PUL and scattered systems is that the former has genes related to alginate degradation located in a more concentrated area, with alginate lyase, substrate-binding proteins, and alginate-specific TonB-dependent receptor genes clustered together to form an alginate utilization locus. In contrast, the genes encoding alginate lyases in the scattered system are usually distributed throughout the genome [22].

Comparative analysis revealed that the alginate metabolism pathway of HZ11 conforms to the characteristics of PUL. An AUL gene cluster existed in the genome, with 50% of the predicted alginate lyase genes located in this AUL. When transporting polymeric substrates from the extracellular space to the periplasm space, susC and susD play essential roles. These two proteins bind to the cell membrane, forming a “pedal bin” model [40]. The presence of susC and susD is a typical marker of the Gram-negative bacteria PUL. However, we did not find the susD gene in the genome of HZ11. Xu et al. also reported that susD was absent in 11 strains of *Pseudoalteromonas* when the AUL of these bacteria was studied [41]. Thomas et al. reported that susD is a protein unique to the order Alteromonadales [42]. The Proteobacteria order acquired the alginate utilization system from Alteromonadales through horizontal gene transfer but lost susD [43].

In most microbial genomes, the number of CAZymes genes is less than 2% of the genes encoding proteins. Even in the genomes of microorganisms considered carbohydrate degraders, it seldom exceeds 5% [44]. Among the *M.* sp. strains involved in this study, the highest proportion of CAZyme gene was found in *M. thermocotolerans* DAU221, accounting for 7.01%. The lowest was *M. variant* ATCC 700307, with only 1.92%. The number of CAZyme genes in HZ11 accounted for 4.57%. Furthermore, there were 825 coding genes related to carbohydrate transport and metabolism in the HZ11 genome. This information suggests that HZ11 has an excellent ability to degrade carbohydrates [45].

In addition to the PL and GH genes, CBM modules often play important roles in polysaccharide degradation. CBMs support enzymes in binding to target substrates, especially insoluble polysaccharides, thereby reducing the distance between the enzyme and the substrate and promoting enzymatic activity [40,46]. The alginate lyase belongs to the PL6, PL7, PL17 and PL38 families, and some alginate lyase genes contain the CBM16 or CBM32 domain, which are important carbohydrate-binding proteins. Several studies have shown that CBM16 and CBM32 domains have specific effects on the enzymatic characteristics, substrate recognition, and product structure of alginate lyases [47,48,49]. This impact could be positive or negative. The specific details need to be further verified through experiments. CBM6 is attached to enzymes with different substrate specificities, such as xylanase, cellulase, agarase, and laminarinase, and has binding specificity for xylan, cellulose, agar, and chitin [50,51]. In the HZ11 genome, the GH16, GH86, and GH5 family genes related to agar and cellulose hydrolysis contain the CBM6 domain.

Brown algae are considered promising biomass for producing ethanol. However, the alginates in brown algae are difficult for traditional ethanol fermentation microorganisms to utilize, resulting in low ethanol yields and challenges for bioconversion [52]. Microorganisms that can utilize alginate lack the ethanol biosynthesis pathway [53]. Genetically engineered strains were constructed for brown algae fermentation. However, these engineered strains require high oxygen levels during growth, necessitating the regulation of oxygen content in the reactor to achieve an optimal balance between ethanol accumulation and cell growth [54]. Sawabe et al. discovered a *Vibrio halioticoli* strain in abalone guts that could convert alginate into acetic acid. However, due to an imbalance in the reductive power, it could not further convert acetic acid into ethanol [55,56].

The HZ11 genome contains metabolic pathways for various carbohydrates or sugar alcohols such as alginate, mannitol, and laminarin, which can generate pyruvate through the ED pathway and further convert it into ethanol. The results show that HZ11 has the potential to utilize alginates, or even brown algae, as substrates for fermentation to produce ethanol. Its ability to produce ethanol and the fermentation conditions require more detailed investigation.

## 4. Materials and Methods

### 4.1. Screening and Identification of Microbulbifer sp. HZ11

The seawater samples were collected from the Zhoushan Islands of the East China Sea, and the method of selection and separation was described by Sun et al. [57]. The diluted seawater was spread on marine basal culture medium solid plates containing 0.5% sodium alginate (sodium alginate 5 g/L, (NH_4_)_2_SO_4_ 5 g/L, K_2_HPO_4_ 2 g/L, NaCl 30 g/L, MgSO_4_·7H_2_O 1 g/L, FeSO_4_·7H_2_O 0.01 g/L, agar 15 g/L, pH 7.5) and incubated upside down for 3 d [58]. After the plates were soaked for 30 min in 1 M calcium chloride solution, the colonies surrounded by zones of hydrolysis were identified as alginate lyase bacteria [59].

HZ11 was cultured in 2216E medium (peptone, 5 g/L; yeast extract, 1 g/L; sea salt, 33.3 g/L; ferric phosphate, 0.01 g/L) at 28 °C and 120 rpm for 24 h. The cell pellet was collected by centrifugation at 12,000 rpm for 2 min, and the DNA was extracted according to the instructions of the FastPure Bacteria DNA Isolation Mini Kit (Vazyme, Nanjing, China). The 16S rRNA gene was amplified via the universal primers 27F (5′-AGAGTTTGATCCTGGCTCAG-3′) and 1492R (5′-GGTTACCTTGTTACGACTT-3′). The PCR amplification products were subsequently sequenced by Sangon. The sequencing results were submitted for comparison to the NCBI database.

MEGA 7.0.26 was used to construct a maximum likelihood (ML) phylogenetic tree to confirm the evolutionary position of HZ11 [60]. The sequence information used for the tree construction is listed in Table S2. The tree-building model included GTR+I+G, Gamma 1, and 1000 bootstrap replications. *Marinimicrobium koreense* M9T was used as an outgroup [20].

### 4.2. Phenotypic Characteristics

The strain HZ11 was inoculated into marine basal culture medium containing 0.5% alginate. The effects of different temperatures (4, 15, 20, 25, 28, 30, 37, 41, 45, 50, and 55 °C), different pH values (3.0–10.0), and different concentrations of sodium chloride (20–90 g/L) on strain HZ11 were explored to assess its growth range, optimal temperature, optimal pH value, and optimal concentration of sodium chloride [11].

HZ11 was isolated by streaking on 2216E solid plates and incubating at 30 °C for 48 h. The morphology of a single colony of HZ11 was observed. HZ11 cells were stained with Gram stain solution (BKMAMLAB, Changde, China) and examined under a microscope. The physiological and biochemical identification of the target strain was carried out according to the “Handbook of Common Bacterial System Identification” [61], including the citrate utilization test, indole production test, nitrate reduction test, and catalase activity test.

### 4.3. Genome Sequencing and Annotation

HZ11 was cultured, the cell pellet was collected using Method 4.1, and the genome sequencing work was completed by Shanghai Majorbio Bio-pharm Technology Co., Ltd. (Shanghai, China). The sequencing and annotation process was performed according to Huang et al. [28]. Other genome sequences were obtained from NCBI (Table S3). The ANI Calculator (https://www.ezbiocloud.net/tools/ani, accessed on 1 June 2024) was used to calculate the average nucleotide identity (ANI) [62]. The digital DNA–DNA hybridization (dDDH) was calculated using the Genome-to-Genome Distance Calculator 3.0 (http://ggdc.dsmz.de/ggdc.php, accessed on 31 July 2024) [63].

### 4.4. The Utilizing Abilities of Carbohydrates

The CAZymes and carbohydrate-binding modules were predicted using the NCBI CDD (https://www.ncbi.nlm.nih.gov/Structure/cdd/wrpsb.cgi, accessed on 18 June 2024), CAZy database (http://www.cazy.org/, accessed on 20 June 2024), and dbCAN3 server (https://bcb.unl.edu/dbCAN2/blast.php, accessed on 20 June 2024) [64,65,66]. The metabolic pathways of HZ11 for alginate and other carbohydrates were constructed based on the Kyoto Encyclopedia of Genes and Genome (KEGG) database and the dbCAN—PUL database (http://aca.unl.edu/dbCAN_PUL/dbCAN_PUL/blast, accessed on 22 June 2024) [67,68].

To verify whether HZ11 could degrade agar, 40 μL of logarithmic-phase HZ11 bacterial suspension was added in 4 equal drops to the surface of the solid plates containing 1.5% agar in marine basal culture medium, and incubated at 30 °C in a temperature incubator for 48 h. To verify whether HZ11 could degrade starch, the solid plates were supplemented with 1% (*w*/*v*) soluble starch into the above-covered culture. The plates were stained with 10 mL of Lugol’s iodine solution for 30 min after the culture. To verify whether HZ11 could degrade cellulose, the solid plates were supplemented with 1% (*w*/*v*) carboxymethylcellulose sodium into the culture described above. The plates were stained with 10 mL of 0.1% (*w*/*v*) Congo red solution for 30 min and then decolorized with 10 mL of 1 M sodium chloride solution for 15 min. A clear hydrolysis zone around the colonies after incubation or staining indicated that HZ11 can degrade the corresponding polysaccharides. These methods refer to Muhammad et al. [69].

In the marine basal culture medium, different sugars or alcohols were added separately to study the ability of HZ11 to utilize different carbon sources, including polysaccharides (starch, cellulose, alginate, and fucoidan), disaccharides (trehalose, sucrose, lactose, and maltose), monosaccharides (glucose, rhamnose, fructose, xylose, galactose, and mannose) and alcohols (xylitol, glycerol, mannitol, and sorbitol), with final concentrations of 1%. HZ11 suspension cultures were shaken at 120 rpm and 28 °C for 38 h. Each carbon source was tested in 3 parallel experiments. The OD_600_ was measured, and the ability of the strain to utilize the tested carbohydrates as a carbon source for growth was determined.

### 4.5. Detection of Alginate Lyase Activity and Enzymatic Properties

At a 2% inoculation rate, the logarithmic-phase HZ11 bacterial suspension was inoculated into the enzyme-producing medium (alginic acid 17 g/L, urea 1 g/L, K_2_HPO_4_ 2 g/L, NaCl 55 g/L, MgSO_4_·7H_2_O 1 g/L, FeSO_4_·7H_2_O 0.01 g/L, pH 7.5) at 28 °C and 80 rpm for 38 h. In the low-temperature state, the HZ11 culture medium was centrifuged at 10,000 rpm for 3 min, and the supernatant was collected and filtered through a 0.22 μm membrane to obtain the crude enzyme. The protein concentration in the fermentation mixture was detected via a BCA reagent kit (Vazyme, China).

The enzymatic properties of the HZ11 alginate lyase were studied. The experimental method was adapted from Kikuchi et al. and adjusted according to the actual situation [70]. The primary substrate consisted of 0.5% (*w*/*v*) alginate, 50 mM Na-phosphate, and 0.3 M sodium chloride at pH 7.5. The enzyme and substrate were mixed evenly and incubated at 30 °C for 30 min, after which 200 μL of the reaction mixture was mixed with 150 μL of 3, 5-dinitrosalicylic acid (DNS) reagent and boiled for 10 min. The reducing sugar concentration was calculated on the basis of the absorbance at 540 nm using glucose as a standard. One enzyme activity unit (U) was the amount of alginate lyase required to produce 1 μg of reduced sugar within 1 min (mL) [45].

The reaction mixture was incubated at different temperatures (15–70 °C) to determine the optimal temperature. For the optimal pH, the enzymes were mixed with different pH substrates (Na-acetate 5.0–6.0; Na-phosphate 6.0–7.5; HEPES–NaOH 7.5–8.5; glycine–NaOH 8.5–9.5; 0.1 M). The concentration of sodium chloride in the substrate was set to 0–0.5 M to determine the optimal NaCl concentration. Different metal ions were added to the reaction mixture at a final concentration of 5 mM to determine the effect of the metal ions on the enzyme activity. The substrate specificity of HZ11 alginate lyase was determined using alginate, polyM and polyG (0.5%, *w*/*v*) as substrates. For thermal stability, the enzyme was incubated at different temperatures (30, 40 and 50 °C) for different durations (0–4 h) and cooled on ice. For pH stability, the enzyme was incubated in different buffers at 4 °C for 24 h, after which the residual enzyme activity was detected.

The enzymatic reaction kinetics were determined under optimal reaction conditions. The enzyme was mixed with substrates (alginate/polyM/polyG 0.1%, 0.2%, 0.4%, 0.6%, 0.8%, 1.0% and 1.2% (*w*/*v*), glycine 0.1 M, and sodium chloride 0.1 M, pH 8.5) and incubated at 50 °C for 15 min.

### 4.6. Preparation and Detection of the Enzymatic Degradation of Alginate

Crude enzyme and the substrate (alginate/polyM/polyG 1.5% (*w*/*v*), 50 mM Na-phosphate, and 0.3 M sodium chloride, pH 7.5) were mixed evenly. Changes in the reducing sugar content were detected in the reaction system after 0, 2, 6, 12, 24, 36, 48 and 60 h of incubation at 30 °C.

The TLC method was used to determine the enzymatic degradation patterns of alginate, polyM, and polyG. The developing solvent was n-butanol/acetic acid/water = 3:2:3. The staining reagent was ethanol/concentrated sulfuric acid 9:1 (*v*/*v*), and the samples were heated at 110 °C for 5 min after staining [71].

### 4.7. RT-qPCR of Alginate Lyase Genes

The gene expression levels of alginate lyases were detected via RT–qPCR [72]. The logarithmic-phase HZ11 bacterial suspension was added to marine basal culture medium containing 0.5% glucose or 0.5% alginate at a 2% inoculation rate. The cultures were incubated at 120 rpm and 28 °C in a shaking incubator for 24 h. Three parallel sets of each carbon source were used. The bacterial RNA was extracted via the Bacterial RNA Extraction Kit (Vazyme, China), and the extracted RNA was reverse-transcribed via HiScript II Q RT SuperMix for qPCR (+gDNA wiper) (Vazyme, China) to obtain template cDNA. The genes to be detected and the amplification primers are listed in Table S4, with the housekeeping gene *pyrG* used as an internal reference gene. The qPCR amplification system included 1 μL of template, 5 μL of ChamQ Blue Universal SYBR qPCR Master Mix (Vazyme, China), 0.5 μL of forward primer, 0.5 μL of reverse primer, and 3 μL of ddH_2_O. The amplification program was as follows: preincubation at 95 °C for 5 min; 3-step amplification at 95 °C for 10 s, 55 °C for 15 s, and 72 °C for 10 s; and 40 cycles.

## 5. Conclusions

This study identified and classified the polysaccharide lyase-producing strain HZ11 as a *Microbulbifer* sp. The assembled genome was approximately 4,248,867 bp long, with a GC content of 56.68%. A total of 3537 genes were predicted, including 3477 protein-coding genes, 50 tRNAs and 9 rRNA sequences. The strain HZ11 contained 159 CAZymes, including 11 AA, 31 CBM, 12 CE, 56 GH, 25 GT and 24 PL. HZ11 can produce alginate lyase, agarase, amylase, and cellulase, and it can grow using various carbohydrates or alcohols as carbon sources. The alginate lyases of HZ11 can cleave alginate, polyM and polyG to produce AOSs, with maximum activity at 50 °C and pH 8.5. The TLC results revealed that the products of the enzymatic hydrolysis of HZ11 were mainly monosaccharides or AOSs with DPs of 2–3. In conclusion, these results indicate that HZ11 provides a new potential source for polysaccharide-degrading enzymes and oligosaccharide preparations.

## Figures and Tables

**Table 1:** Physiological and biochemical characteristics of strain HZ11.

Iterms	Results	Iterms	Results
Growth temperature (optimal)	15–50 °C (28–30 °C)	Citrate utilization	+
Growth pH (optimal)	3.0–9.5 (8.5)	Urease	+
Growth NaCl concentration (optimal)	20–90 g/L (70 g/L)	Tryptophan deaminase	−
Ornithine decarboxylase	−	Indole production	−
Lysine decarboxylase	−	Oxidase	+
Arginine decarboxylase	−	Nitrate reduction	+
Arginine dihydroenzyme	+	Catalase	+

Note: +: positive reaction; −: negative reaction.

**Table 2:** Genomic characterization of HZ11.

Category	Number
Sequence	1
Genome size (bp)	4,248,867
Bases (bp)	171,101,961
Total reads	21,909
Coverage (%)	100
N50 (bp)	8400
C+G%	56.68
Total ncRNA	74
Repeats Sequence	84
Protein-coding genes	3477
Total genes predicted	3537

**Table 3:** Multiple genes related to polysaccharide degradation in the genome of strain HZ11.

Catabolic Enzymes	Family	Gene ID	EC Number
Alginate lyase	PL18	gene0070	4.2.2.-
	PL7	gene0073, gene0074, gene0628, gene1910, gene1911, gene3508	
	PL38	gene0297	
poly(β-D-mannuronate) lyase	PL6	gene0081	4.2.2.3
oligo-alginate lyase	PL17	gene0080	4.2.2.26
α-Amylase	GH13	gene1263, gene3041, gene3042, gene3044, gene3048	3.2.1.1
Cellulase	GH5	gene1049, gene1101, gene2339	3.2.1.4
	GH9	gene1102	
Endo-1,4-β-xylanase	GH10	gene2337	3.2.1.8
Chitinase	GH18	gene1079, gene1080, gene2143, gene2144	3.2.1.14
α-glucosidase	GH13	gene2990	3.2.1.20
β-Glucosidase	GH3	gene2341, gene2345, gene3054	3.2.1.21
β-Galactosidase	GH2	gene0299	3.2.1.23
	GH167	gene2256	
Pullulanase	GH13	gene0349	3.2.1.41
beta-N-acetyl hexosaminidase	GH20	gene0682, gene2447, gene2985	3.2.1.52
	GH3	gene1932, gene2984	
β-agarase	GH50	gene2235, gene2236, gene2277	3.2.1.81
	GH16	gene2279	
	GH86	gene2283	
neopullulanase	GH13_46	gene3042	3.2.1.135
endo-chitodextinase	GH108	gene2542	3.2.1.202
Pectate lyase	PL1	gene0313, gene2373	4.2.2.2
	PL10	gene0316, gene0699	

**Table 4:** Enzymatic kinetic parameters of alginate lyase.

	polyM	polyG	Alginate
Km (mg mL^−1^)	6.95 ± 0.15	9.53 ± 0.23	5.95 ± 0.12
Vmax (U mL^−1^ min^−1^)	294.8 ± 17.08	231.0 ± 21.11	494.8 ± 26.22

## Data Availability

https://www.ncbi.nlm.nih.gov/genbank/ accessed on 1 June 2024.

## Supplementary Materials

The following supporting information can be downloaded at https://www.mdpi.com/article/10.3390/md22120569/s1; Table S1: dDDH values between HZ11 and other *Microbulbifer* genus strain; Table S2: 16S rRNA sequences from all the selected species for phylogenetic analysis; Table S3: The genomic information of *Microbulbifer* genus strains; Table S4. Primers used for qRT-PCR.

## References

[1] Beuder S., Braybrook S. (2023). Brown algal cell walls and development. Semin. Cell. Dev. Biol..

[2] Chi S., Liu T., Wang X.M., Wang R., Wang S.S., Wang G.L., Shan G.L., Liu C. (2018). Functional genomics analysis reveals the biosynthesis pathways of important cellular components (alginate and fucoidan) of Saccharina. Curr. Genet..

[3] Cheng D.Y., Jiang C.C., Xu J.C., Liu Z., Mao X.Z. (2020). Characteristics and applications of alginate lyases: A review. Int. J. Biol. Macromol..

[4] Liu J., Yang S.Q., Li X.T., Yan Q.J., Reaney M., Jiang Z.Q. (2019). Alginate Oligosaccharides: Production, Biological Activities, and Potential Applications. Compr. Rev. Food Sci. Food Saf..

[5] Wang X.X., Xu W., Dai Q.Y., Liu X.Y., Guang C., Zhang W., Mu W. (2023). Characterization of a thermostable PL-31 family alginate lyase from *Paenibacillus ehimensis* and its application for alginate oligosaccharides bioproduction. Enzym. Microb. Technol..

[6] Qiu X.M., Qi L., Zheng B.D., Zhao W.L., Ye J., Xiao M.T. (2023). Preparation and potential antitumor activity of alginate oligosaccharides degraded by alginate lyase from *Cobetia marina*. Carbohydr. Res..

[7] Falkeborg M., Cheong L.Z., Gianfico C., Sztukiel K., Kristensen K., Glasius M., Xu X.B., Guo Z. (2014). Alginate oligosaccharides: Enzymatic preparation and antioxidant property evaluation. Food Chem..

[8] Zhu Y.B., Wu L.Y., Chen Y.H., Ni H., Xiao A.F., Cai H.N. (2016). Characterization of an extracellular biofunctional alginate lyase from marine *Microbulbifer* sp. ALW1 and antioxidant activity of enzymatic hydrolysates. Microbiol. Res..

[9] Wang H., Zhu B.W. (2024). Directed preparation of algal oligosaccharides with specific structures by algal polysaccharide degrading enzymes. Int. J. Biol. Macromol..

[10] Perumal P., Dong C.D., Chauhan A., Anisha G., Kadri M., Chen C.W., Singhania R., Patel A. (2023). Advances in oligosaccharides production from algal sources and potential applications. Biotechnol. Adv..

[11] Zheng Y.T., Wang Y.J., Dan M.L., Li Y.P., Zhao G.H., Wang D.M. (2023). Characterization of degradation patterns and enzymatic properties of a novel alkali-resistant alginate lyase AlyRm1 from *Rubrivirga marina*. Curr. Res. Food Sci..

[12] Li L., Zhu B.W., Yao Z., Jiang J.J. (2023). Directed preparation, structure-activity relationship and applications of alginate oligosaccharides with specific structures: A systematic review. Food Res. Int..

[13] Zhu B., Yin H. (2015). Alginate lyase: Review of major sources and classification, properties, structure-function analysis and applications. Bioengineered.

[14] Wang P., Cai Y., Zhong H., Chen R.T., Yi Y.T., Ye Y.R., Li L.L. (2024). Expression and characterization of an efficient alginate lyase from *Psychromonas* sp. SP041 through metagenomics analysis of rotten kelp. Genes.

[15] Silva M., Silva C., Santos L., Medeiros J., Vieira R., Abud A., Almeida R., Tonholo J. (2022). Alginate lyase produced by filamentous fungus through solid state fermentation using *Sargassum* from the Brazilian coast. Waste Biomass Valorization.

[16] Amin M., Bolch C., Adams M., Burke C. (2017). Isolation of alginate lyase-producing bacteria and screening for their potential characteristics as abalone probionts. Aquac. Res..

[17] Mohapatra B.R. (2022). Fermentation Medium Optimization, Molecular modelling and docking analysis of the alginate lyase of a novel *Pseudomonas* Sp. LB56 isolated from seaweed waste. Biotechnol. Biotechnol. Equip..

[18] Zhou H.X., Xu S.S., Yin X.J., Wang F.L., Li Y. (2020). Characterization of a new bifunctional and cold-adapted Polysaccharide Lyase (PL) family 7 alginate lyase from *Flavobacterium* sp.. Mar. Drugs.

[19] Xue Z., Sun X.M., Chen C., Zhang X.Y., Chen X.L., Zhang Y.Z., Fan S.J., Xu F. (2022). A novel alginate lyase: Identification, characterization, and potential application in alginate trisaccharide preparation. Mar. Drugs.

[20] Huang H.Q., Mo K.L., Hu Y.H., Liu M., Zhu J., Zou X.X., Bao S.X. (2020). *Microbulbifer harenosus* sp. nov., an alginate-degrading bacterium isolated from coastal sand. Int. J. Syst. Evol. Microbiol..

[21] Wang X.H., Zhang Y.Q., Zhang X.R., Zhang X.D., Sun X.M., Wang X.F., Sun X.H., Song X.Y., Zhang Y.Z., Wang N. (2024). High-level extracellular production of a trisaccharide-producing alginate lyase AlyC7 in *Escherichia coli* and its agricultural application. Mar. Drugs.

[22] Zhang L.Z., Li X., Zhang X.Y., Li Y.J., Wang L.S. (2021). Bacterial alginate metabolism: An important pathway for bioconversion of brown algae. Biotechnol. Biofuels.

[23] Long L.F., Hu Q.S., Wang X.X., Li H.B., Li Z.P., Jiang Z.D., Ni H., Li Q.B., Zhu Y.B. (2022). A bifunctional exolytic alginate lyase from Microbulbifer sp. ALW1 with salt activation and calcium-dependent catalysis. Enzym. Microb. Technol..

[24] Li H.B., Huang X.Y., Yao S.X., Zhang C.H., Hong X., Wu T., Jiang Z.D., Ni H., Zhu Y.B. (2022). Characterization of a bifunctional and endolytic alginate lyase from *Microbulbifer* sp. ALW1 and its application in alginate oligosaccharides production from *Laminaria japonica*. Protein Expr. Purif..

[25] Jiang J., Jiang Z.Q., Yan Q.J., Han S.S., Yang S.Q. (2024). Biochemical characterization of a novel bifunctional alginate lyase from *Microbulbifer arenaceous*. Protein Expr. Purif..

[26] Chen C., Li X., Lu C., Zhou X., Chen L., Qiu C., Jin Z., Long J. (2024). Advances in alginate lyases and the potential application of enzymatic prepared alginate oligosaccharides: A mini review. Int. J. Biol. Macromol..

[27] Hifney A., Gomaa M., Fawzy M., Abdel K. (2019). Optimizing a low-cost production process of crude fucoidanase by *Dendryphiella arenaria* utilizing *Cystoseira trinodis* (Phaeophyceae) and enzymatic hydrolysis of the brown algal biomass. Waste Biomass Valorization.

[28] Huang H.Q., Zheng Z.G., Zou X.X., Wang Z.X., Gao R., Zhu J., Hu Y.H., Bao S.X. (2022). Genome analysis of a novel polysaccharide-degrading bacterium *Paenibacillus algicola* and determination of alginate lyases. Mar. Drugs.

[29] Li X., Yang M., Mo K.L., Hu Y.H., Gu H.J., Sun D.M., Bao S.X., Huang H. (2024). Genome analysis of multiple polysaccharide-degrading bacterium *Microbulbifer thermotolerans* HB226069: Determination of alginate lyase activity. Mar. Biotechnol..

[30] Zhong W.M., Agarwal V. (2024). Polymer degrading marine *Microbulbifer* bacteria: An un(der)utilized source of chemical and biocatalytic novelty. Beilstein J. Org. Chem..

[31] Li Z., Du Z., Li H., Chen Y., Zheng M., Jiang Z., Du X., Ni H., Zhu Y. (2022). Characterisation of marine bacterium *Microbulbifer* sp. ALW1 with *Laminaria japonica* degradation capability. AMB Express.

[32] Wakabayashi M., Sakatoku A., Noda F., Noda M., Tanaka D., Nakamura S. (2012). Isolation and characterization of *Microbulbifer* species 6532A degrading seaweed thalli to single cell detritus particles. Biodegradation.

[33] Rekha P., Shastry R., Athmika N. (2023). Genomic potential for exopolysaccharide production and differential polysaccharide degradation in closely related *Alteromonas* sp. PRIM-21 and *Alteromonas fortis* 1^T^. Antonie van Leeuwenhoek.

[34] Nguyen T., Vuong T., Han H., Li Z., Lee Y., Ko J., Nedashkovskaya O.I., Kim S.G. (2023). Three marine species of the genus *Fulvivirga*, rich sources of carbohydrate-active enzymes degrading alginate, chitin, laminarin, starch, and xylan. Sci. Rep..

[35] Yoon J., Kim H., Kang K., Oh T., Park Y. (2003). Transfer of *Pseudomonas elongata* Humm 1946 to the genus *Microbulbifer* as *Microbulbifer elongatus* comb. nov. Int. J. Syst. Evol. Microbiol..

[36] Park S., Yoon S., Ha M., Yoon J. (2017). *Microbulbifer aestuariivivens* sp. nov., isolated from a tidal flat. Int. J. Syst. Evol. Microbiol..

[37] Moh T., Furusawa G., Amirul A. (2017). *Microbulbifer aggregans* sp. nov., isolated from estuarine sediment from a mangrove forest. Int. J. Syst. Evol. Microbiol..

[38] Calabrese S., Mohanty B.P., Malik A.A. (2022). Soil microorganisms regulate extracellular enzyme production to maximize their growth rate. Biogeochemistry.

[39] Jiang Z., Guo Y., Wang X., Li H., Ni H., Li L., Xiao A., Zhu Y. (2019). Molecular cloning and characterization of AlgL17, a new exo-oligoalginate lyase from *Microbulbifer* sp. ALW1. Protein Expr. Purif..

[40] Wardman J., Bains R., Rahfeld P., Withers S. (2022). Carbohydrate-active enzymes (CAZymes) in the gut microbiome. Nat. Rev. Microbiol..

[41] Xu F., Cha Q.Q., Zhang Y.Z., Chen X.L. (2021). Degradation and utilization of alginate by marine Pseudoalteromonas: A Review. Appl. Environ. Microbiol..

[42] Thomas F., Hehemann J., Hehemann J., Rebuffet E., Rebuffet E., Czjzek M., Michel G. (2011). Environmental and gut Bacteroidetes: The food connection. Front. Cell. Infect. Microbiol..

[43] Thomas F., Barbeyron T., Tonon T., Génicot S., Czjzek M., Michel G. (2012). Characterization of the first alginolytic operons in a marine bacterium: From their emergence in marine *Flavobacteriia* to their independent transfers to marine *Proteobacteria* and human gut *Bacteroides*. Environ. Microbiol..

[44] Mann A., Hahnke R., Huang S.X., Werner J., Xing P., Barbeyron T., Huettel B., Stüber K., Reinhardt R., Harder J. (2013). The Genome of the alga-associated marine Flavobacterium *Formosa agariphila* KMM 3901T reveals a broad potential for degradation of algal polysaccharides. Appl. Environ. Microbiol..

[45] Cheng W.W., Yan X.Y., Xiao J.L., Chen Y.Y., Chen M.H., Jin J.Y., Bai Y., Wang Q., Liao Z.Y., Chen Q.Z. (2020). Isolation, identification, and whole genome sequence analysis of the alginate-degrading bacterium *Cobetia* sp. cqz5-12. Sci. Rep..

[46] Mei X.W., Liu G.C., Chen G.N., Zhang Y.Y., Xue C.H., Chang Y.G. (2024). Characterization and structural identification of a family 16 carbohydrate-binding module (CBM): First structural insights into porphyran-binding CBM. Int. J. Biol. Macromol..

[47] Liu H., Huang M., Wei S.X., Wang X.W., Zhao Y.Q., Han Z.Y., Ye X.F., Li Z.K., Ji Y.L., Cui Z.L. (2024). Characterization of a multi-domain exo-β-1, 3-galactanase from *Paenibacillus xylanexedens*. Int. J. Biol. Macromol..

[48] Mei X.W., Chang Y.G., Shen J.J., Zhang Y.Y., Xue C.H. (2020). Expression and characterization of a novel alginate-binding protein: A promising tool for investigating alginate. Carbohydr. Polym..

[49] Xu H.Y., Gao Q., Li L., Su T., Ming D.M. (2024). How alginate lyase produces quasi-monodisperse oligosaccharides: A normal-mode-based docking and molecular dynamics simulation study. Carbohydr. Res..

[50] Michel G., Barbeyron T., Kloareg B., Czjzek M. (2009). The family 6 carbohydrate-binding modules have coevolved with their appended catalytic modules toward similar substrate specificity. Glycobiology.

[51] Norlander S., Jasilionis A., Allahgholi L., Wennerberg C., Grey C., Adlercreutz P., Karlsson E. (2024). Inter domain linker region affects properties of CBM6 in GH5_34 arabinoxylanases and alters oligosaccharide product profile. Glycobiology.

[52] Zhang W., Ren X.H., Bao L.Y. (2018). Enzyme detection and metabolic process tracking of ethanol fermentation by a natural alginate fermentation strain. Braz. Arch. Biol. Technol..

[53] Dharshini R.S., Fathima A.A., Dharani S.R., Ramya M. (2020). Utilization of alginate from brown macroalgae for ethanol production by *Clostridium phytofermentans*. Appl. Biochem. Microbiol..

[54] Zhang W., Mao Y.Q., Liu Z.W., Wang M.J. (2021). Ethanol production from *Colpomenia sinuosa* by an alginate fermentation strain *Meyerozyma guilliermondii*. Indian J. Microbiol..

[55] Sawabe T., Setoguchi N., Inoue S., Tanaka R., Ootsubo M., Yoshimizu M., Ezura Y. (2003). Acetic acid production of *Vibrio halioticoli* from alginate: A possible role for establishment of abalone-*V. halioticoli* association. Aquaculture.

[56] Inohara Y., Chunqi J., Mino S., Swabe T. (2023). A first marine *Vibrio* biocatalyst to produce ethanol from alginate, which is a rich polysaccharide in brown macroalgal biomass. Curr. Microbiol..

[57] Sun X.K., Gong Y., Shang D.D., Liu B.T., Du Z.J., Chen G.J. (2022). Degradation of alginate by a newly isolated marine bacterium *Agarivorans* sp. B2Z047. Mar. Drugs.

[58] Dou W.F., Wei D., Li H., Li H., Rahman M., Shi J., Xu Z.H., Ma Y.H. (2013). Purification and characterisation of a bifunctional alginate lyase from novel *Isoptericola halotolerans* CGMCC 5336. Carbohydr. Polym..

[59] Hisano T., Nishimura M., Yamashita T., Imanaka T., Muramatsu T., Kimura A., Murata K. (1994). A simple method for determination of substrate specificity of alginate lyases. J. Ferment. Bioeng..

[60] Kumar S., Stecher G., Tamura K. (2016). MEGA7: Molecular Evolutionary Genetics Analysis Version 7.0 for Bigger Datasets. Mol. Biol. Evol..

[61] Dong X.Z., Cai M.Y. (2001). Handbook of Identification of Common Bacterial System.

[62] Chalita M., Kim Y., Park S., Oh H., Cho J., Moon J., Baek N., Moon C., Lee K., Yang J. (2024). EzBioCloud: A genome-driven database and platform for microbiome identification and discovery. Int. J. Syst. Evol. Microbiol..

[63] Meier-Kolthoff J.P., Sardà C.J., Peinado-Olarte R.L., Göker M. (2022). TYGS and LPSN: A database tandem for fast and reliable genome-based classification and nomenclature of prokaryotes. Nucleic Acids Res..

[64] Wang J.Y., Chitsaz F., Derbyshire M., Gonzales N., Gwadz M., Lu S.N., Marchler G., Song J., Thanki N., Yamashita R. (2023). The conserved domain database in 2023. Nucleic Acids Res..

[65] Drula E., Garron M., Dogan S., Lombard V., Henrissat B., Terrapon N. (2022). The carbohydrate-active enzyme database: Functions and literature. Nucleic Acids Res..

[66] Zheng J.F., Ge Q.W., Yan Y.C., Zhang X.P., Huang L., Yin Y.B. (2023). dbCAN3: Automated carbohydrate-active enzyme and substrate annotation. Nucleic Acids Res..

[67] Kanehisa M., Sato Y., Kawashima M. (2022). KEGG mapping tools for uncovering hidden features in biological data. Protein Sci..

[68] Ausland C., Zheng J.F., Yi H.D., Yang B.W., Li T., Feng X.H., Zheng B., Yin Y.B. (2021). dbCAN-PUL: A database of experimentally characterized CAZyme gene clusters and their substrates. Nucleic Acids Res..

[69] Muhammad N., Avila F., Nedashkovskaya O., Kim S. (2023). Three novel marine species of the genus *Reichenbachiella* exhibiting degradation of complex polysaccharides. Front. Microbiol..

[70] Kikuchi M., Konno N., Suzuki T., Fujii Y., Kodama Y., Isogai A., Habu N. (2020). A bacterial endo-β-1, 4-glucuronan lyase, CUL-I from *Brevundimonas* sp. SH203, belonging to a novel polysaccharide lyase family. Protein Expr. Purif..

[71] Meng Q., Zhou L.C., Hassanin H., Jiang B., Liu Y.C., Chen J.J., Zhang T. (2021). A new role of family 32 carbohydrate binding module in alginate lyase from *Vibrio natriegens* SK42.001 in altering its catalytic activity, thermostability and product distribution. Food Biosci..

[72] He X.X., Zhang Y.H., Wang X.L., Zhu X.Y., Chen L.R., Liu W.Z., Liu Q.Q., Ran L.M., Cheng H.J., Zhang X.H. (2022). Characterization of multiple alginate lyases in a highly efficient alginate-degrading *Vibrio* strain and its degradation strategy. Appl. Environ. Microbiol..

